# Performance of the Pooled Cohort Equations to Estimate Atherosclerotic Cardiovascular Disease Risk by Body Mass Index

**DOI:** 10.1001/jamanetworkopen.2020.23242

**Published:** 2020-10-29

**Authors:** Rohan Khera, Ambarish Pandey, Colby R. Ayers, Mercedes R. Carnethon, Philip Greenland, Chiadi E Ndumele, Vijay Nambi, Stephen L. Seliger, Paulo H. M. Chaves, Monika M. Safford, Mary Cushman, Vanessa Xanthakis, Vasan S. Ramachandran, Robert J. Mentz, Adolfo Correa, Donald M. Lloyd-Jones, Jarett D. Berry, James A. de Lemos, Ian J. Neeland

**Affiliations:** 1 Section of Cardiovascular Medicine, Department of Internal Medicine, Yale School of Medicine, New Haven, Connecticut; 2 Center for Outcomes Research and Evaluation, Yale New Haven Hospital, New Haven, Connecticut; 3 Division of Cardiology, University of Texas Southwestern Medical Center, Dallas; 4 Department of Preventive Medicine, Northwestern University Feinberg School of Medicine, Chicago, Illinois; 5 Department of Medicine, Johns Hopkins University School of Medicine, Baltimore, Maryland; 6 Michael E. DeBakey VA Medical Center, Baylor College of Medicine, Houston, Texas; 7 Department of Medicine, University of Maryland School of Medicine, Baltimore; 8 Benjamin Leon Center for Geriatric Research and Education, Florida International University, Miami; 9 Division of General Internal Medicine, Department of Medicine, Weill Cornell Medical College, New York, New York; 10 Department of Medicine, University of Vermont, Burlington; 11 Department of Internal Medicine, Boston University School of Medicine, Boston, Massachusetts; 12 Duke Clinical Research Institute, Durham, North Carolina; 13 Department of Medicine, University of Mississippi Medical Center, Jackson; 14 Department of Medicine, University Hospitals Cleveland Medical Center, Cleveland, Ohio; 15 Case Western Reserve University School of Medicine, Cleveland, Ohio

## Abstract

**Importance:**

Obesity is a global health challenge and a risk factor for atherosclerotic cardiovascular disease (ASVCD). Performance of the pooled cohort equations (PCE) for ASCVD risk by body mass index (BMI; calculated as weight in kilograms divided by height in meters squared) is unknown.

**Objective:**

To assess performance of the PCE across clinical BMI categories.

**Design, Setting, and Participants:**

This cohort study used pooled individual-level data from 8 community-based, prospective, longitudinal cohort studies with 10-year ASCVD event follow-up from 1996 to 2016. We included all adults ages 40 to 79 years without baseline ASCVD or statin use, resulting in a sample size of 37 311 participants. Data were analyzed from August 2017 to July 2020.

**Exposures:**

Participant BMI category: underweight (<18.5), normal weight (18.5 to <25), overweight (25 to <30), mild obesity (30 to <35), and moderate to severe obesity (≥35).

**Main Outcomes and Measures:**

Discrimination (Harrell *C* statistic) and calibration (Nam-D'Agostino χ^2^ goodness-of-fit test) of the PCE across BMI categories. Improvement in discrimination and net reclassification with addition of BMI, waist circumference, and high-sensitivity C-reactive protein (hsCRP) to the PCE.

**Results:**

Among 37 311 participants (mean [SD] age, 58.6 [11.8] years; 21 897 [58.7%] women), 380 604 person-years of follow-up were conducted. Mean (SD) baseline BMI was 29.0 (6.2), and 360 individuals (1.0%) were in the underweight category, 9937 individuals (26.6%) were in the normal weight category, 13 601 individuals (36.4%) were in the overweight category, 7783 individuals (20.9%) were in the mild obesity category, and 5630 individuals (15.1%) were in the moderate to severe obesity category. Median (interquartile range [IQR]) 10-year estimated ASCVD risk was 7.1% (2.5%-15.4%), and 3709 individuals (9.9%) developed ASCVD over a median (IQR) 10.8 [8.5-12.6] years. The PCE overestimated ASCVD risk in the overall cohort (estimated/observed [E/O] risk ratio, 1.22; 95% CI, 1.18-1.26) and across all BMI categories except the underweight category. Calibration was better near the clinical decision threshold in all BMI groups but worse among individuals with moderate or severe obesity (E/O risk ratio, 1.36; 95% CI, 1.25-1.47) and among those with the highest estimated ASCVD risk ≥20%. The PCE *C* statistic overall was 0.760 (95% CI, 0.753-0.767), with lower discrimination in the moderate or severe obesity group (*C* statistic, 0.742; 95% CI, 0.721-0.763) compared with the normal-range BMI group (*C* statistic, 0.785; 95% CI, 0.772-0.798). Waist circumference (hazard ratio, 1.07 per 1-SD increase; 95% CI, 1.03-1.11) and hsCRP (hazard ratio, 1.07 per 1-SD increase; 95% CI, 1.05-1.09), but not BMI, were associated with increased ASCVD risk when added to the PCE. However, these factors did not improve model performance (*C* statistic, 0.760; 95% CI, 0.753-0.767) with or without added metrics.

**Conclusions and Relevance:**

These findings suggest that the PCE had acceptable model discrimination and were well calibrated at clinical decision thresholds but overestimated risk of ASCVD for individuals in overweight and obese categories, particularly individuals with high estimated risk. Incorporation of the usual clinical measures of obesity did not improve risk estimation of the PCE. Future research is needed to determine whether incorporation of alternative high-risk obesity markers (eg, weight trajectory or measures of visceral or ectopic fat) into the PCE may improve risk prediction.

## Key Points

**Question:**

What is the performance of the pooled cohort equations (PCE) for estimation of atherosclerotic cardiovascular disease (ASCVD) risk by body mass index?

**Findings:**

In this pooled analysis of 8 longitudinal cohort studies that included 37 311 adults, the PCE demonstrated acceptable model discrimination but significantly overestimated risk of atherosclerotic cardiovascular disease in individuals with higher body mass index, with better calibration near clinical decision thresholds and less optimal calibration for the groups at highest risk. Incorporation of usual clinical measures of obesity did not result in more accurate risk estimation compared with standard PCE.

**Meaning:**

These findings suggest that the PCE could be used as a risk-estimation tool to guide prevention and treatment strategies in adults across clinical BMI categories, but may overestimate risk of ASCVD for individuals in overweight and obese categories.

## Teaser

This cohort study evaluates the performance of the pooled cohort equations in estimating the risk of atherosclerotic cardiovascular disease risk by body mass index range.

## Introduction

The pooled cohort equations (PCE) were introduced in 2013 as sex- and race-specific tools for estimating 10-year absolute rates of atherosclerotic cardiovascular disease (ASCVD) events in a primary prevention population.^1^ The risk estimates were derived based on a combination of established cardiovascular risk factors examined prospectively in specific cohorts selected for derivation of the PCE.^1^ Variables included in the PCE are age, sex, race (ie, White, Black, or other), smoking status, systolic blood pressure, hypertension treatment status, diabetes status, and total and high-density lipoprotein (HDL) cholesterol levels.^1,2^ Risk estimates are then used to guide recommendations for preventive therapies (eg, lifestyle modification, statin medication, and antihypertensive therapies).^3,4^ Given the use of the PCE estimates for risk-stratification and risk-modification strategies, it is critical to ensure that the equations perform adequately in diverse at-risk groups. However, when applied to diverse population samples, the performance of the PCE has varied, with evidence of acceptable calibration in broad clinical populations but overestimation in some and underestimation in other selected groups.^5,6^ The use of the PCE for individuals with obesity has not been adequately studied but has important implications for ASCVD prevention.

Individuals with obesity represent a high-risk group that constitutes approximately 35% of the US adult population.^7,8,9^ While obesity is associated with alterations in cardiovascular risk factors and increased risk for cardiovascular events, some of the event risk may not be fully explained by the risk factor burden captured in the PCE.^10^ Furthermore, the rate of observed events among some subsets of individuals with obesity may be lower than among individuals with weights in the normal range, a finding frequently referred to as the obesity paradox.^11^ In addition, the cohorts used to derive the PCE had a lower proportion of individuals with obesity compared with contemporary populations, potentially limiting the generalizability of the PCE among individuals with obesity. Finally, PCE risk estimates may be enhanced by including the severity or pattern of obesity (eg, abdominal obesity), neither of which are currently captured in the PCE.

To address these knowledge gaps, we combined risk factor and incident ASCVD data from 8 large community-based cohort studies, including 5 contemporary cohorts not used in derivation of the PCE, to compare discrimination and calibration of the PCE among individuals across categories for body mass index (BMI; calculated as weight in kilograms divided by height in meters squared). We additionally examined the effects of adding obesity-related measures to the PCE for ASCVD risk estimation.

## Methods

### Data Sources

Protocols for this cohort study were approved by the institutional review boards at each participating institution. All participants provided written informed consent. The study followed the Strengthening the Reporting of Observational Studies in Epidemiology (STROBE) reporting guideline. We analyzed pooled individual-level data from 8 longitudinal cohort studies: Atherosclerosis Risk in Communities (ARIC),^12^ Coronary Artery Risk Development in Young Adults (CARDIA),^13^ Cardiovascular Health Study (CHS),^14^ Dallas Heart Study (DHS),^15^ Framingham Heart Study (FHS) Third Generation cohort,^16^ Jackson Heart Study (JHS),^17^ Multi-Ethnic Study of Atherosclerosis (MESA),^18^ and Reasons for Geographic and Racial Differences in Stroke (REGARDS).^19^ The design and selection criteria for these longitudinal cohorts have been described previously.^12,13,14,15,16,17,18,19^ Data from the most contemporary follow-up assessment in each cohort with a corresponding baseline assessment from at least 10 years previously was used to evaluate the estimated risk and observed rates of ASCVD. Data from earlier examination cycles of 3 of these cohorts (ie, ARIC,^12^ CARDIA,^13^ CHS^14^) were used in the original derivation of the PCE. Nearly 30% of individuals sampled in ARIC are also part of the JHS but were included only once as part of the ARIC cohort in this study.^17^

### Study Population

To match the methods and guideline-based recommendations for the use of the PCE, we included all adults aged 40 to 79 years with a BMI measurement who, at the time of BMI assessment, were free from established ASCVD, defined as a prior history of coronary artery disease (ie, angina or myocardial infarction), cerebrovascular disease (ie, stroke or transient ischemic attack), or peripheral vascular disease. Participants were sequentially excluded if at the time of assessment of BMI and risk factor exposure, they (1) had a low-density lipoprotein (LDL) cholesterol level of less than 70 mg/dL or 190 mg/dl or higher (to convert to micromoles per liter, multiply by 0.0259), (2) were using a statin or other lipid-lowering medication (eg, niacin, fibrates, or bile acid binding–resins), (3) were missing data on any of the variables necessary to calculate PCE risk estimate, or (4) were missing follow-up data. Of the cohorts used in the original derivation, this study included 9744 individuals: 6336 individuals from ARIC,^12^ 2771 from CARDIA,^13^ and 637 from CHS^14^; of the cohorts not used in the original derivation, this study included 27 567 individuals: 2214 from DHS,^15^ 2524 from FHS Third Generation,^16^ 3287 from JHS,^17^ 5249 from MESA,^18^ and 14293 from REGARDS^19^ (eFigure 1 in the Supplement). Baseline and follow-up dates were recorded for all studies included in the analysis (eTable 1 in the Supplement).

### Variables and Outcomes

Variables included self-reported age, sex, race (ie, White, Black, or other), diabetes status, smoking status, antihypertensive medication use, total cholesterol and HDL cholesterol levels, and systolic blood pressure. We also obtained information on BMI, waist circumference (data missing for 0.7% of individuals), and high-sensitivity C-reactive protein (hsCRP, data missing for 10.3% of individuals) at the index visit. (Some study cohorts provided data that was already redacted, so only percentages are available for missing data.) Data on use of statin or other cholesterol-lowering medication after the index visit was collected in 5 study cohorts (data not available in ARIC, CHS, or JHS). All covariates were measured using similar approaches,^12,13,14,15,16,17,18,19^ and variable definitions were harmonized for analytical purposes.

The primary outcome of the study was 10-year risk of ASCVD, defined as the risk of coronary heart disease death, nonfatal myocardial infarction, or fatal or nonfatal ischemic stroke within 10 years of the first visit. In all included cohorts, study outcomes were determined based on standardized adjudication by an expert panel. Detailed descriptions of event-ascertainment procedures and adjudication processes in these cohorts have been previously published.^12,13,14,15,16,17,18,19^

### Statistical Analysis

First, we created a pooled data set of all eligible individuals from each cohort and stratified these individuals by BMI, based on World Health Organization classification of BMI: underweight (<18.5), normal weight (18.5 to <25), overweight (25 to <30), mild obesity (30 to <35), and moderate to severe obesity (≥35).^20^ Next, for all participants, we estimated the 10-year risk of ASCVD using the unmodified PCE (ie, without refitting of β coefficients to the current data set) and data on age, sex, race, blood total cholesterol and HDL cholesterol concentrations, systolic blood pressure, use of antihypertensive therapy, diabetes status, and smoking status.^1^ For this analysis, individuals with the race designation other were treated as White.

Trends in characteristics were evaluated across BMI categories, in the overall sample, and by specific cohort using trend tests for continuous variables and Cochran-Armitage test for categorical variables. Estimated 10-year ASCVD risk was compared with observed event rates, overall and by BMI category, using several metrics. Model discrimination in each BMI category was assessed using Harrell *C* statistic, with 0.7 or greater considered acceptable.^21,22^ We then categorized all individuals into risk groups based on their estimated 10-year risk of ASCVD: low (<5%), borderline (5% to <7.5%), intermediate (7.5% to <20%), or high (≥20%).^23^ We next generated plots of observed vs estimated ASCVD risk for each BMI category stratified by the guideline-based risk categories, as well as by deciles of risk. Participants without ASCVD events were censored at their last available follow-up or at time of non-ASCVD death through 10 years.

We used a Cox proportional hazards model for time-to-event analyses, and the proportional hazards assumption was satisfied for all BMI categories. Calibration was assessed from these plots both visually and using the Nam-D'Agostino χ^2^ goodness-of-fit test; a nonsignificant χ^2^ (*P* > .05) indicated good calibration.^24^ We calculated expected-to-observed (E/O) risk ratios (RRs) with corresponding 95% CIs using bootstrapping (1000 repeated iterations each) across BMI categories. An RR of 1 indicated perfect calibration across the full spectrum of risk, while RRs greater than 1 indicated mean overestimation of risk and RRs less than 1 indicated mean underestimation of risk. Model calibration by BMI categories was also determined in subgroups defined by race/ethnicity (Black and non-Black [White and other races]), sex, and age at the first study visit (to control for secular trends in obesity in the population due to time-varying cohort baselines). To account for potential model optimism, model discrimination and calibration were assessed across BMI categories separately in the 3 cohorts used to derive the PCE (ie, ARIC, CARDIA, and CHS) and 5 other more contemporary cohorts (ie, studies initiated after 2000) that were not part of the derivation of the PCE (ie, DHS, FHS, JHS, MESA, and REGARDS). Finally, we explored the addition of a priori–defined measures of obesity and inflammation (ie, BMI, waist circumference, and hsCRP) to a model with the PCE refit to the current data set with incident ASCVD as the outcome and reported the hazard ratio (HR) and its 95% CI for each additional obesity covariate. We evaluated the distribution of these covariates to justify their inclusion as linear terms in the model (eAppendix in the Supplement).

We then compared model discrimination using changes in model *C* statistics compared with the baseline model using nonparametric bootstrapping to calculate 95% CIs and the net reclassification improvement (theoretical range, −2 to 2) after adding the obesity metrics. We also assessed likelihood ratios across obesity classes and cohorts to assess and compare overall model fit (eAppendix in the Supplement). These steps were repeated as sensitivity analyses by (1) estimating 10-year risk of ASCVD using PCE variables recalibrated for the current data set, (2) excluding individuals with statin or lipid-lowering therapy initiated after the baseline visit, and (3) replacing the lipid profile with BMI or waist circumference in the PCE instead of adding the anthropometric variables to the lipid profile to assess modified, nonlaboratory PCE risk estimation. We also performed a competing risks analysis by treating non-ASCVD deaths as competing events in time-to-event analyses according to the methods of Fine and Gray.^25^ All statistical analyses were performed between August 2017 and July 2020 using SAS statistical software version 9.4 (SAS Institute). *P* value tests were 2-sided, and *P* values less than 0.05 were considered statistically significant.

## Results

### Cohort Characteristics

Of 37 311 participants without preexisting ASCVD, with mean (SD) age 58.6 (11.8) years, 21 897 (58.7%) were women, 14 291 individuals (38.3%) were Black, 20 745 individuals (55.6%) were White, and 2 275 individuals (6.1%) were other race. The median (interquartile range [IQR]) follow-up in the total cohort was 10.8 (8.5 to 12.6) years, with a total of 380 604 person-years of follow-up.

The mean (SD) BMI in the overall population sample was 29.0 (6.2), with 360 individuals [1.0%] in the underweight category, 9937 individuals [26.6%] in the normal weight range, 13 601 individuals (36.4%) in the overweight category, 7783 individuals (20.9%) in the mildly obese category, and 5630 individuals (15.1%) in the moderately to severely obese category. The characteristics of participants at baseline, overall and by BMI category, were recorded (Table 1), as well as characteristics by specific cohort (eTable 1 in the Supplement). Participant characteristics varied across BMI categories, and compared with individuals in the underweight and normal weight range categories, those in the overweight and obese categories were younger and more likely to be women, be Black, and have ASCVD risk factors (ie, hypertension, diabetes, and lower HDL cholesterol concentration) (Table 1). Fewer individuals in the obese category were active smokers.

**Table 1.** Characteristics of Study Participants by BMI Categories

Characteristic	Participants, No. (%)						*P* value for trend
	Overall (N = 37 311)	BMI					
		<18.5 (n = 360)	18.5 to <25 (n = 9937)	25 to <30 (n = 13 601)	30 to <35 (n = 7783)	≥35 (n = 5630)	
Age, mean (SD), y	58.6 (11.8)	60.3 (14.1)	59.3 (12.5)	59.4 (11.7)	58.4 (11.1)	56.1 (11.1)	<.001
Men	15 414 (41.3)	103 (28.6)	3809 (38.3)	6812 (50.1)	3298 (42.4)	1392 (24.7)	<.001
Black race	14 291 (38.3)	109 (30.3)	2482 (25.0)	4770 (35.1)	3588 (46.1)	3342 (59.4)	<.001
Systolic BP, mean (SD), mm Hg	125 (18)	118 (21)	120 (19)	125 (17)	128 (17)	130 (18)	<.001
Cardiovascular risk factors							
Hypertension	13 108 (35.1)	69 (19.2)	2256 (22.7)	4467 (32.8)	3342 (42.9)	2974 (52.8)	<.001
Diabetes	4091 (11.0)	14 (3.9)	468 (4.7)	1225 (9.0)	1153 (14.8)	1231 (21.9)	<.001
Smoking	5415 (14.5)	123 (34.2)	1777 (17.9)	1888 (13.9)	974 (12.5)	653 (11.6)	<.001
Total cholesterol, mean (SD), mg/dL	198 (31)	194 (31)	197 (31)	199 (31)	199 (31)	196 (31)	.48
HDL cholesterol, mean (SD), mg/dL	53 (16)	67 (17)	60 (18)	52 (15)	49 (14)	49 (13)	<.001
Waist circumference, mean (SD), cm	96.5 (15.4)	70.8 (8.7)	82.5 (8.9)	94.3 (8.8)	104.2 (9.8)	117.7 (13.9)	<.001
hsCRP, median (IQR), mg/dL	0.2 (0.1-0.4)	0.1 (0-0.2)	0.1 (0-0.3)	0.2 (0.1-0.4)	0.3 (0.1-0.5)	0.4 (0.2-0.9)	<.001
Estimated 10-y ASCVD risk, median (IQR), %	7.1 (2.5-15.4)	6.4 (1.4-17.2)	5.8 (1.6-14.7)	7.8 (2.9-16.5)	8.1 (3.3-16.3)	7.0 (2.7-14.8)	<.001
Observed 10-y ASCVD event rates	3709 (9.9)	37 (10.3)	912 (9.2)	1460 (10.7)	814 (10.5)	486 (8.6)	.57
CHD death	582 (1.5)	10 (2.8)	145 (1.5)	215 (1.5)	103 (1.3)	109 (1.9)	.29
Nonfatal myocardial infarction	1527 (4.1)	10 (2.8)	378 (3.8)	622 (4.6)	337 (4.3)	180 (3.2)	.26
Fatal or nonfatal stroke	1600 (4.3)	17 (4.7)	389 (3.9)	623 (4.6)	374 (4.8)	197 (3.5)	.72

Abbreviations: ASCVD, atherosclerotic cardiovascular disease; BMI, body mass index (calculated as weight in kilograms divided by height in meters squared); BP, blood pressure; CHD, coronary heart disease; HDL, high-density lipoprotein; hsCRP, high-sensitivity C-reactive protein; IQR, interquartile range.

SI conversion factors: To convert HDL and total cholesterol to millimoles per liter, multiply by 0.0259; to convert hsCRP to milligrams per liter, multiply by 10.

### Estimated and Observed Outcomes

For the 10-year estimated ASCVD risk for the overall cohort based on the PCE, mean (SD) was 11.0% (11.7%) and median (IQR) was 7.1% (2.5% to 15.4%). Among 3709 individuals (9.9%) who developed ASCVD over a follow-up period of 10.8 years, 582 individuals (15.7%) died from coronary heart disease, 1528 individuals (41.2%) had a first nonfatal myocardial infarction, and 1599 individuals (43.1%) had a fatal or first nonfatal stroke. Across all BMI categories, median (IQR) PCE-estimated 10-year risk of ASCVD ranged between 5.8% (1.6%-14.7%) for the normal weight category and 8.1% (3.3%-16.3%) for the mildly obese category, whereas observed 10-year ASCVD event incidence during follow-up ranged between 486 individuals (8.6%) in the moderate to severe obesity category and 1460 individuals (10.7%) in the overweight category. Observed incidence was 912 individuals (9.2%) in the normal weight category (Table 1).

### PCE Model Performance

The PCE demonstrated acceptable discrimination for the entire study population, with a Harrell *C* statistic of 0.760 (95% CI, 0.753-0.767) (Table 2). Discrimination of the PCE for ASCVD was lower in overweight and obese categories than in the normal BMI range category (Table 2). The PCE overestimated risk of ASCVD across all BMI categories except the underweight category (eg, normal weight: E/O RR, 1.21; 95% CI, 1.14-1.28; mild obesity: E/O RR, 1.24; 95% CI, 1.17-1.32; and moderate to severe obesity: E/O RR, 1.36; 95% CI, 1.25-1.47) (Table 2), and overestimation was greatest for the moderate to severe obesity category (E/O RR, 1.36; 95% CI,1.25-1.47]). Overestimation of risk was most notable at higher estimated ASCVD risk (≥20%), and calibration near the clinical decision threshold of 7.5% was visually better in all BMI groups (Figure). Calibration results were similar when stratified by clinically meaningful risk groups (ie, <5%, 5 to <7.5%, 7.5 to 20%, and ≥20%) (Figure and eTable 2 in the Supplement) and across deciles of estimated risk (eFigure 2 in the Supplement). Likelihood ratio statistics were produced for the models, overall and in BMI- and cohort-based subgroups (eTable 3 in the Supplement). Results were similar when PCE component variables were refit for the current data set.

**Table 2.** Discrimination and Calibration of the Pooled Cohort Equations Across Body Mass Index Categories

BMI category	*C* statistic (95% CI)			Mean E/O RR^a^ (95% CI)		
	All cohorts	Derivation cohort studies^b^	Nonderivation cohort studies^c^	All cohorts	Derivation cohort studies^b^	Nonderivation cohort studies^c^
Total	0.760 (0.753-0.767)	0.758 (0.746-0.770)	0.761 (0.752-0.770)	1.22 (1.18-1.26)	0.97 (0.92-1.02)	1.32 (1.28-1.36)
Underweight (<18.5)	0.789 (0.731-0.848)	0.777 (0.651-0.903)	0.793 (0.727-0.859)	1.01 (0.79-1.35)	0.69 (0.41-1.58)	1.09 (0.84-1.55)
Normal weight (18.5 to <25)	0.785 (0.772-0.798)	0.777 (0.755-0.799)	0.792 (0.777-0.807)	1.21 (1.14-1.28)	0.87 (0.79-0.96)	1.36 (1.27-1.45)
Overweight (25 to <30)	0.759 (0.748-0.770)	0.758 (0.739-0.777)	0.759 (0.745-0.773)	1.17 (1.12-1.22)	0.94 (0.87-1.01)	1.28 (1.21-1.35)
Mild obesity (30 to <35)	0.738 (0.722-0.755)	0.744 (0.718-0.770)	0.734 (0.713-0.755)	1.24 (1.17-1.32)	1.03 (0.93-1.16)	1.32 (1.21-1.43)
Moderate to severe obesity (≥35)	0.742 (0.721-0.763)	0.737 (0.697-0.777)	0.739 (0.715-0.764)	1.36 (1.25-1.47)	1.23 (1.03-1.47)	1.38 (1.26-1.51)

Abbreviations: BMI, body mass index (calculated as weight in kilograms divided by height in meters squared); E/O RR, expected-to-observed risk ratio.

^a^ An RR of 1 indicates perfect calibration across the full spectrum of risk; greater than 1, overestimation of risk; and less than 1, underestimation of risk.

^b^ Atherosclerosis Risk in Communities, Coronary Artery Risk Development in Young Adults, and Cardiovascular Health Study.

^c^ Dallas Heart Study, Framingham Heart Study Third Generation, Jackson Heart Study, Multi-Ethnic Study of Atherosclerosis, and Reasons for Geographic and Racial Differences in Stroke.

**Figure.**
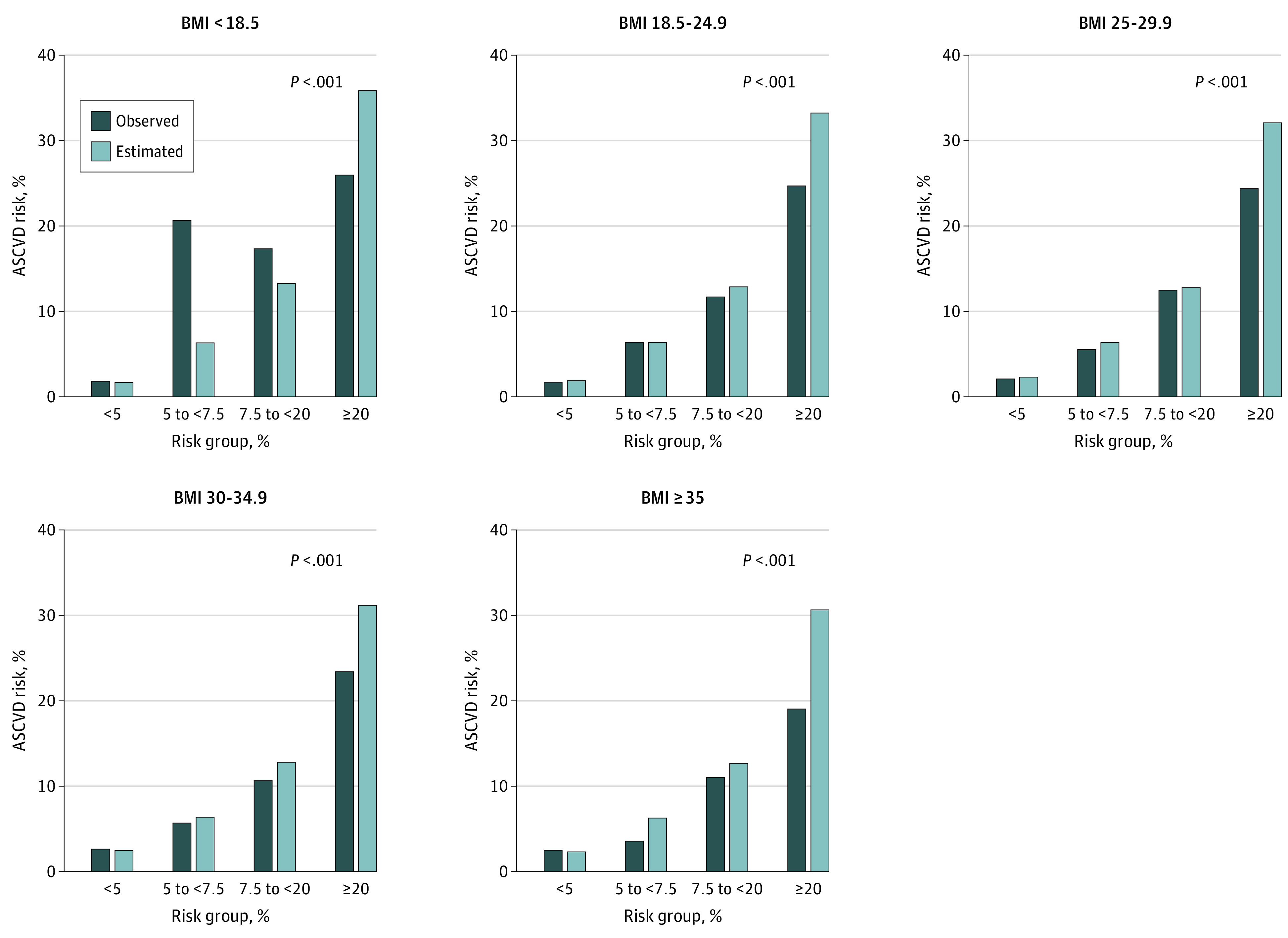
Calibration by Body Mass Index (BMI) Category Rates of events estimated by the pooled cohort equations compared with rates of events observed in the study, across subgroups based on estimated risk, by BMI category. *P* values are for Nam-D'Agostino χ^2^ goodness-of-fit test; a nonsignificant χ^2^ (*P* > .05) indicates good calibration. ASCVD indicates atherosclerotic cardiovascular disease. BMI calculated as weight in kilograms divided by height in meters squared.

In sensitivity analyses stratified by sex and race, results were generally similar, although calibration was worse among men compared with women in the mild obesity category (E/O RR, 1.39; 95% CI, 1.28-1.52 vs E/O RR, 1.09; 95% CI, 1.01-1.21) and moderate to severe obesity category (E/O RR, 1.52; 95% CI, 1.32-1.77 vs E/O RR, 1.30; 95% CI, 1.17-1.43]) and among Black individuals compared with White individuals in the mild obesity category (E/O RR, 1.31; 95% CI, 1.20-1.43 vs E/O RR, 1.18; 95% CI, 1.09-1.29) (eTable 4 in the Supplement). In analyses stratified by involvement in development of the PCE (ie, derivation vs non-derivation cohorts), PCE discrimination was similar and calibration was better in derivation cohorts compared with nonderivation cohorts (Table 2). In sensitivity analyses, results were similar when stratified based on age of participants at index visit (ie, older vs younger than median) and after excluding individuals with statin or lipid-lowering therapy use initiated after baseline visit (eTable 5 in the Supplement). The proportion of statin or lipid-lowering therapy initiation after baseline was similar across BMI categories in this study (21% in the normal weight range, 22% in the overweight category, and 23% in the obese category).

Finally, we assessed whether performance of the PCE would be improved by including obesity-related measures (ie, BMI, waist circumference, and hsCRP as continuous variables). We found that waist circumference and hsCRP, but not BMI, were individually associated with an increased hazard of ASCVD overall (waist circumference: HR, 1.07; 95% CI, 1.03 to 1.11; hsCRP: HR, 1.07; 95% CI, 1.05 to 1.09) (Table 3) and in nearly all race and sex groups (eTable 6 in the Supplement). There was no improvement in *C* statistic with addition of any of these measures individually to the other PCE variables, either in the overall study cohort or separately in derivation and nonderivation cohorts (Table 3) or in race- and sex-specific subgroups (eTable 6 in the Supplement). Addition of the 3 measures together led to statistically significant improvement in model discrimination vs standard PCE (*C* statistic, 0.764; 95% CI, 0.757 to 0.771; *P* = .001 vs *C* statistic, 0.760; 95% CI, 0.753 to 0.767; *P* = .001). Associations between obesity-related measures and ASCVD were similar in a competing risks model used in place of a traditional Cox model (eTable 7 in the Supplement). Results were similar when BMI, waist circumference, or hsCRP was used in place of, rather than in addition to, lipid profile variables in the PCE model (eTable 8 in the Supplement). BMI and waist circumference did not result in significant net reclassification improvement (NRI) at the 7.5% ASCVD risk threshold, and NRI with hsCRP was significant (NRI, 0.003; *P* = .01) (eTable 9 in the Supplement). There were no significant improvements in continuous NRI for the measures individually or in combination (BMI: NRI, −0.091; 95% CI, −0.262 to 0.080; waist circumference: NRI, 0.009; 95% CI, −0.330 to 0.349; hsCRP: NRI, 0.098; 95% CI, 0.017 to 0.180; combined: NRI, 0.069; 95% CI, −0.058 to 0.196).

**Table 3.** Association Of Obesity-Related Measures With Atherosclerotic Cardiovascular Disease Events

Measure	All cohorts		Derivation cohort studies^a^		Nonderivation cohort studies^b^	
	*C* statistic (95% CI)^c^	*P* value	C statistic (95% CI)	*P* value	C statistic (95% CI)	*P* value
BMI	1.00 (0.96-1.04)^c^	NA	1.02 (0.95-1.09)^c^	NA	0.98 (0.94-1.03)^c^	NA
Waist circumference	1.07 (1.03-1.11)^c^	NA	1.06 (0.99-1.13)^c^	NA	1.08 (1.03-1.13)^c^	NA
hsCRP	1.07 (1.05-1.09)^c^	NA	(1.08-1.19)^c^	NA	1.06 (1.03-1.09)^c^	NA
Model discrimination						
Overall (standard PCE model)	0.760 (0.753-0.767)		0.758 (0.746-0.770)		0.761 (0.752-0.770)	
Overall + BMI	0.763 (0.756-0.770)	.06	0.760 (0.748-0.772)	.36	0.766 (0.757-0.774)	.23
Overall + waist circumference	0.763 (0.756-0.770)	.88	0.760 (0.748-0.772)	.03	0.766 (0.757-0.774)	.48
Overall + hsCRP	0.762 (0.755-0.769)	.07	0.757 (0.745-0.769)	.99	0.764 (0.756-0.773)	.07
Overall + BMI + waist circumference + hsCRP	0.764 (0.757-0.771)	<.001	0.760 (0.747-0.771)	.003	0.767 (0.759-0.776)	.001

Abbreviations: BMI, body mass index (calculated as weight in kilograms divided by height in meters squared); hsCRP, high sensitivity C-reactive protein; NA, not applicable; PCE, pooled cohort equations.

^a^ Atherosclerosis Risk in Communities, Coronary Artery Risk Development in Young Adults, and Cardiovascular Health Study.

^b^ Dallas Heart Study, Framingham Heart Study Third Generation, Jackson Heart Study, Multi-Ethnic Study of Atherosclerosis, and Reasons for Geographic and Racial Differences in Stroke.

^c^ Hazard ratios per 1-SD change in the exposure, with the addition of each obesity-related measure to a standard pooled cohort equation–based risk-estimation model.

## Discussion

To our knowledge, this is the first large, individual participant–level pooled analysis to assess the performance of the PCE across the full range of BMI categories. This cohort study pooled 8 large, community-based longitudinal cohorts, including nearly 28 000 individuals not included in the derivation of the PCE. The PCE had acceptable model discrimination but were not optimally calibrated and overestimated risk of ASCVD across the range of BMI categories except the underweight category. Calibration was better near the decision threshold in all BMI groups but worse among individuals with moderate to severe obesity and among those with the highest estimated ASCVD risk. The PCE model did not perform as well among men in mild or moderate to severe obesity categories or among Black individuals in the mild obesity category. Furthermore, other obesity-related markers, such as waist circumference and hsCRP, were associated with ASCVD risk independently of the standard PCE model; however, overall model performance was not improved with the addition of variables that captured the degree or pattern of obesity or systemic inflammation or by replacing laboratory-based lipid measures with measures of adiposity.

The PCE have been used in clinical practice for risk-stratification for guiding primary prevention strategies for ASCVD since the equations’ introduction in 2013.^1^ However, researchers have expressed concern about PCE performance in contemporary cohorts, specifically that suboptimal calibration in these cohorts may overestimate risk and lead to unnecessary prescription of statins and implementation of other preventive measures.^5,26^ In addition, given the rising prevalence of obesity in the US and globally,^7^ there is a question whether the PCE perform adequately in more contemporary obese populations given that the equations were derived from population cohorts when obesity was less prevalent. These issues may be most severe in the treatment of individuals with obesity but no metabolic anomalies (up to one-third of individuals with obesity in some studies). In this group, the PCE might underestimate ASCVD risk given that they do not include an obesity-related risk metric.^27^ Alternatively, in some populations, the rate of observed events among individuals with obesity might paradoxically be lower than among individuals in the normal weight range, and the PCE could overestimate risk in these individuals.^28,29^

Our findings addressed these concerns and demonstrated that among individuals with obesity, the PCE performed adequately at the clinical decision risk threshold of 7.5% that is used to define the need for many preventative therapies. However, the models demonstrated miscalibration at higher levels of estimated risk among individuals in overweight and obese categories, where the PCE overestimated risk. Compared with individuals in the normal weight range, those with moderate to severe obesity had a lower observed rate of ASCVD and greater overestimation of risk. This observation may be consistent with an obesity paradox, as has been seen among individuals with prevalent ASCVD. We found that the PCE were less well calibrated in Black individuals with mild obesity. This is a high-risk population with higher rates of stroke, heart failure, and coronary heart disease compared with non-Hispanic White individuals.^30^ Prior studies have suggested that the PCE perform well in Black individuals overall,^31^ although with lower discrimination compared with performance among White individuals.^32^ Our results provide additional insight, especially in light of the substantially higher prevalence of obesity in non-Hispanic Black adults compared with White adults.^33^

Strengths of this study include use of diverse cohorts, both those included and not included in the development of the PCE. This may overcome the issue of model optimism that is a risk if assessment of PCE performance by BMI categories is analyzed only in derivation cohorts. This study is further strengthened by the use of multiple contemporary metrics of model performance, as recommended for assessment of cardiovascular risk in asymptomatic adults.^34,35^ Although we did not study more sophisticated measures of adiposity, we evaluated the impact of adding widely available, clinically applicable obesity-related metrics to the PCE.

This study has several clinical implications. First, our findings suggest that the PCE can be used for ASCVD risk estimation in most individuals with obesity and that it is not necessary to lower PCE risk thresholds for these individuals. Second, the observation that obesity metrics did not substantially improve performance of the PCE model does not exclude the possibility that PCE model performance could be improved by using obesity-related risk factors that more robustly reflect cardiometabolic risk (eg, imaging-based assessments of visceral fat) and that are not uniformly captured in most cohorts. Future studies will be needed to elucidate whether clinical assessment of body fat distribution or alternative biomarkers associated with obesity augment ASCVD risk estimation in contemporary populations. Third, although the additional obesity-related metrics evaluated in our study did not improve performance of the PCE for ASCVD risk estimation, our results should not be misinterpreted to suggest that obesity is benign and unimportant for ASCVD risk assessment. Higher BMI categories were associated with a significant trend for more prevalent ASCVD risk factors. In addition, obesity is clearly associated with excess prevalence of many other adverse health outcomes, and efforts to diagnose and treat it effectively are essential to improving population health. The most recent cholesterol^23^ and primary prevention^36^ guidelines consider metabolic syndrome, a consequence of obesity, a risk-enhancing factor to further inform the discussion regarding statin use in primary prevention.

### Limitations

This study has several limitations. First, issues such as intensive disease screening and medication use after baseline visit, including lipid-lowering agents, could influence ASCVD incidence and lead to miscalibration. However, the proportion of statin and lipid-lowering therapy initiation after baseline was similar across BMI categories in this study, and exclusion of individuals with interim statin or lipid-lowering therapy use did not alter the results. Second, our findings may not be generalizable to individuals in the underweight category, including older populations with a higher prevalence of frailty, given the relatively low sample size of the underweight population. Generalizability of the findings to the broader US population could also be limited by differences in participants making up different cohorts and by evolving care practices over time. Third, our study did not evaluate the impact of physical activity or cardiorespiratory fitness on performance of the PCE in the obese population. However, a 2016 study^29^ found that physically active adults in the overweight or obese categories had similar or lower risks of ASCVD using the PCE compared with inactive adults in the normal weight range. Fourth, outcomes associated with duration of obesity, changes in weight trajectory, visceral adiposity, and other markers of high-risk obesity in the PCE^37^ were not addressed in this study. Fifth, our findings do not generalize to individuals in overweight and obese categories who were beyond the age- and laboratory-range criteria in the standard PCE. Similarly, we classified individuals into Black and non-Black categories for evaluating the PCE based on the approach used in derivation of the PCE. Sixth, the analysis adding obesity-related metrics to the PCE does not account for potential multicollinearity between obesity markers and component risk factors in the model.

## Conclusions

This cohort study found that the PCE had acceptable model discrimination but overestimated risk of ASCVD across the spectrum of BMI, except the underweight category, with better calibration near the decision threshold and less optimal calibration in the highest risk groups. Incorporation of usual clinical measures of obesity did not result in more accurate risk estimation compared with the standard PCE. These findings support the use of the PCE as a risk-estimation tool to guide prevention and treatment strategies in adults regardless of obesity status. Future studies will need to determine whether the use of more specific risk markers for obesity may improve estimation of ASCVD risk among the increasing number of people living with obesity.

## Notes

Supplement. **eAppendix.** Supplemental Methods **eFigure 1.** Study Selection Flowchart **eFigure 2.** Calibration of the Pooled Cohort Equations by Body Mass Index Category **eTable 1.** Characteristics of Study Participants by Study Cohorts **eTable 2.** Number of Events and Individuals Across Obesity Classes by Body Mass Index for Groups Based on Their Estimated 10-Year Risk of Atherosclerotic Cardiovascular Disease from the Pooled Cohort Equations **eTable 3.** Likelihood Ratio Statistic for Model Performance for the Pooled Cohort Equations, Overall and Among Strata Based on Obesity Class and Cohorts **eTable 4.** Calibration of the Pooled Cohort Equations Stratified by Sex and Race **eTable 5.** Sensitivity Analyses **eTable 6.** Association of Obesity-Specific Measures With ASCVD Events in Race- and Sex-Specific Subgroups **eTable 7.** Association Between Obesity-Specific Measures and Incidence of ASCVD in Models That Account for Competing Risk of Non-ASCVD Mortality **eTable 8.** Association of Obesity-Specific Measures With ASCVD Events With Lipid Components Removed From the PCE **eTable 9.** Net Reclassification Improvement With Addition of Obesity-Specific Markers to the PCE Recalibrated to the Data
