# Error in Author Byline

**DOI:** 10.1001/jamanetworkopen.2020.30880

**Published:** 2020-12-02

In the Original Investigation titled “Performance of the Pooled Cohort Equations to Estimate Atherosclerotic Cardiovascular Disease Risk by Body Mass Index,”^1^ published October 29, 2020, author Ramachandran S. Vasan’s name was listed incorrectly. The author’s first and last names were reversed. This article has been corrected.^1^
