# Supplementary material for: Performance of the Pooled Cohort Equations to Estimate Atherosclerotic Cardiovascular Disease Risk by Body Mass Index
Source: JAMA Netw Open. 2020 Oct 29;3(10):e2023242. doi: 10.1001/jamanetworkopen.2020.23242 (PMC7596579; doi:10.1001/jamanetworkopen.2020.23242)
Supplement: Supplement. — eAppendix. Supplemental Methods eFigure 1. Study Selection Flowchart eFigure 2. Calibration of the Pooled Cohort Equations by Body Mass Index Category eTable 1. Characteristics of Study Participants by Study Cohorts eTable 2. Number of Events and Individuals Across Obesity Classes by Body Mass Index for Groups Based on Their Estimated 10-Year Risk of Atherosclerotic Cardiovascular Disease from the Pooled Cohort Equations eTable 3. Likelihood Ratio Statistic for Model Performance for the Pooled Cohort Equations, Overall and Among Strata Based on Obesity Class and Cohorts eTable 4. Calibration of the Pooled Cohort Equations Stratified by Sex and Race eTable 5. Sensitivity Analyses eTable 6. Association of Obesity-Specific Measures With ASCVD Events in Race- and Sex-Specific Subgroups eTable 7. Association Between Obesity-Specific Measures and Incidence of ASCVD in Models That Account for Competing Risk of Non-ASCVD Mortality eTable 8. Association of Obesity-Specific Measures With ASCVD Events With Lipid Components Removed From the PCE eTable 9. Net Reclassification Improvement With Addition of Obesity-Specific Markers to the PCE Recalibrated to the Data [file jamanetwopen-e2023242-s001.pdf]

Supplemental Online Content

Khera R, Pandey A, Ayers CR, et al. Performance of the pooled cohort equations to estimate atherosclerotic cardiovascular disease risk by body mass index. *JAMA Netw Open*. 2020;3(10):e2023242. doi:10.1001/jamanetworkopen.2020.23242

eAppendix. Supplemental Methods

eFigure 1. Study Selection Flowchart

eFigure 2. Calibration of the Pooled Cohort Equations by Body Mass Index Category

eTable 1. Characteristics of Study Participants by Study Cohorts

eTable 2. Number of Events and Individuals Across Obesity Classes by Body Mass Index for Groups Based on Their Estimated 10-Year Risk of Atherosclerotic Cardiovascular Disease from the Pooled Cohort Equations

eTable 3. Likelihood Ratio Statistic for Model Performance for the Pooled Cohort Equations, Overall and Among Strata Based on Obesity Class and Cohorts

eTable 4. Calibration of the Pooled Cohort Equations Stratified by Sex and Race

eTable 5. Sensitivity Analyses

eTable 6. Association of Obesity-Specific Measures With ASCVD Events in Race- and Sex-Specific Subgroups

eTable 7. Association Between Obesity-Specific Measures and Incidence of ASCVD in Models That Account for Competing Risk of Non-ASCVD Mortality

eTable 8. Association of Obesity-Specific Measures With ASCVD Events With Lipid Components Removed From the PCE

eTable 9. Net Reclassification Improvement With Addition of Obesity-Specific Markers to the PCE Recalibrated to the Data

This supplemental material has been provided by the authors to give readers additional information about their work.

© 2020 Khera R et al. *JAMA Network Open*.

eAppendix. Supplemental Methods

Assessment of the distribution of obesity-related variables added to PCE model

We evaluated skewness and kurtosis for body mass index (BMI), waist circumference and high sensitivity C-reactive protein (hsCRP) which were all below 2. Next we, plotted Martingale residuals against the value of the covariate for each subject and fit a LOESS regression to the plot. We then computed the p-value of a Kolmogorov-type supremum test based on a sample of 1,000 simulated residual patterns. For waist circumference, this p-value is 0.339, indicating that all of the 1,000 simulated realizations have an absolute maximum exceeding that of the observed cumulative Martingale residual process. Similarly, for BMI, the corresponding p-values of 0.201 and for hsCRP of 0.132 justified their inclusion as linear terms in our models.

Assessment of likelihood ratios

Parameter estimates in the Cox proportional hazard model were obtained by maximizing the partial likelihood function. Cox and others have shown that this partial log-likelihood can be treated as an ordinary log-likelihood to derive valid (partial) maximum likelihood estimation of the beta coefficients. These likelihood ratios are useful measures of overall model fit, especially when comparing other models. The likelihood function is the product of the probability density functions evaluated at the observed data values.

© 2020 Khera R et al. *JAMA Network Open*.

eFigure 1. Study Selection Flowchart

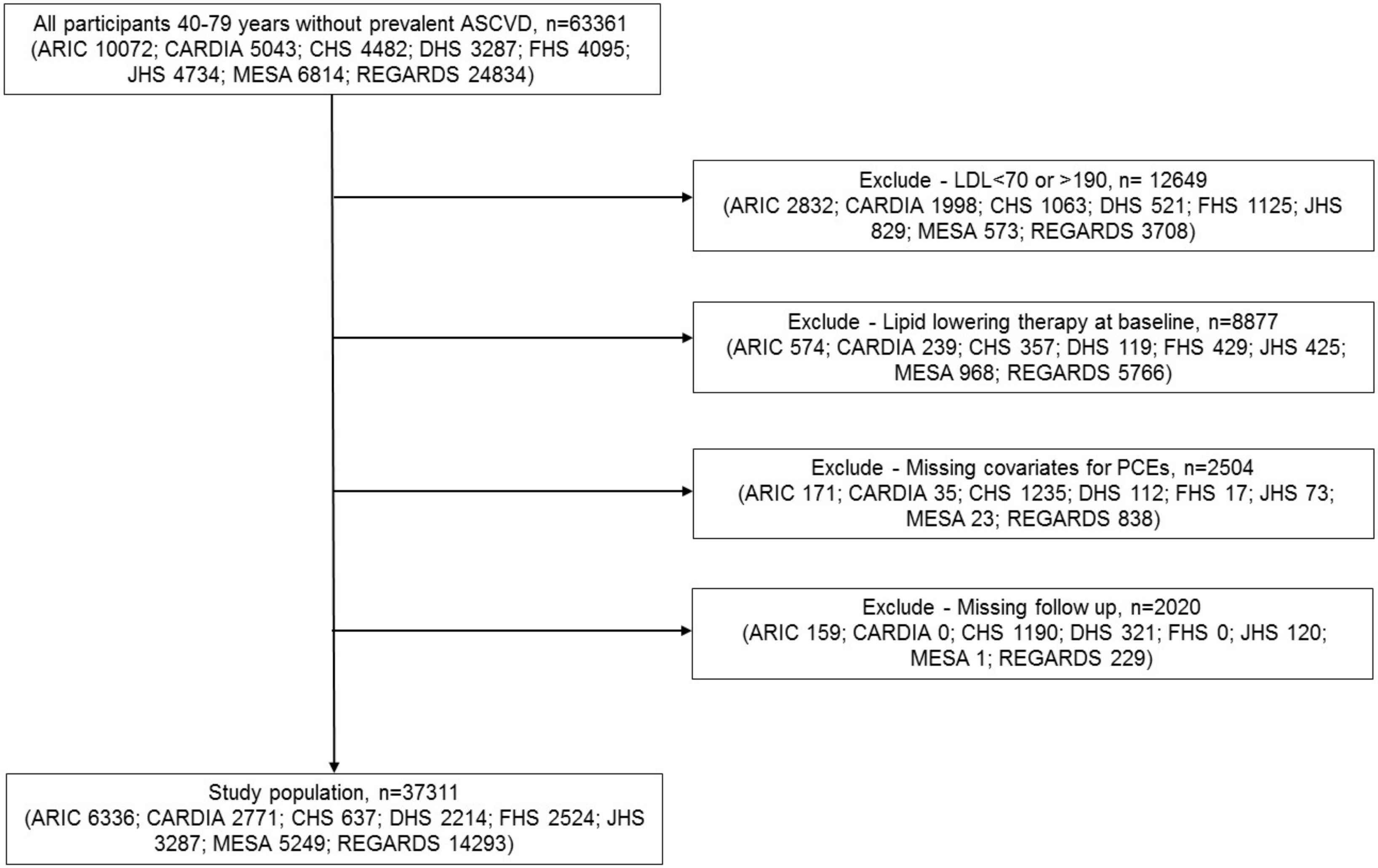

© 2020 Khera R et al. *JAMA Network Open*.

eFigure 2. Calibration of the Pooled Cohort Equations by Body Mass Index Category

Rates of events predicted by the Pooled Cohort Equations compared with rates of events observed in the study, across deciles of predicted risk, by BMI category. P-values are for Nam-D'Agostino χ^2 goodness-of-fit test; a non-significant χ^2 (p-value>0.05) indicates good calibration. ASCVD = atherosclerotic cardiovascular disease, BMI = body mass index.

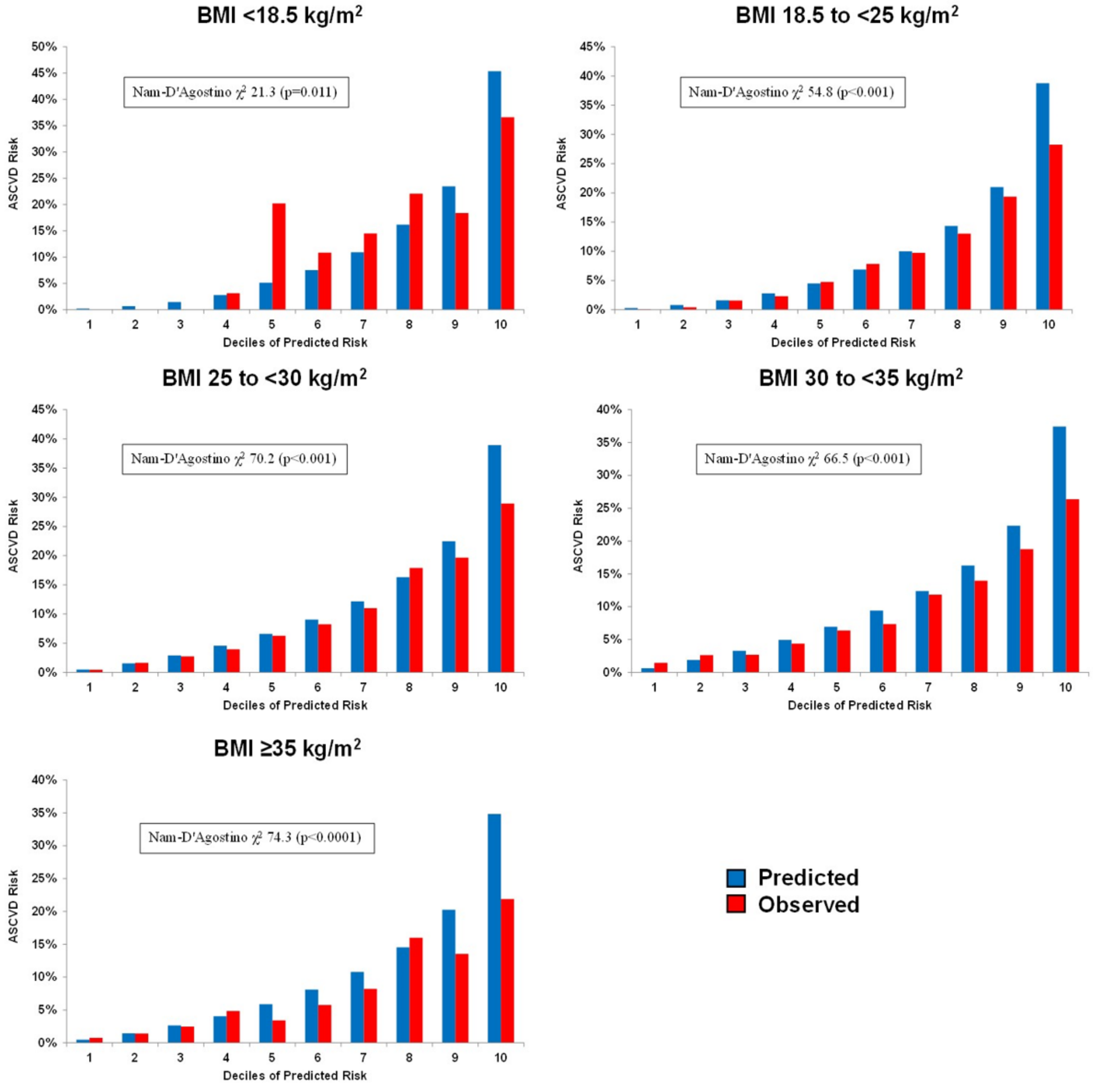

© 2020 Khera R et al. *JAMA Network Open*.

eTable 1. Characteristics of Study Participants by Study Cohorts

Characteristics	Cohorts							
	Derivation cohorts			Non-derivation cohorts				
	**ARIC**	**CARDIA**	**CHS**	**DHS**	**FHS**	**JHS**	**MESA**	**REGARDS**
	6336	2771	637	2214	2524	3287	5249	14293
Study dates	1996-2013	2005-2016	1997-2015	2000-2013	2002-2015	2000-2015	2000-2014	2003-2015
Age, mean (SD), years	63.1 (5.7)	45.1 (3.6)	69.9 (3.7)	43.2 (9.7)	45.9 (8.3)	53.3 (12.8)	61.5 (10.3)	63.6 (9.6)
Men, n (%)	2479 (39.1)	1141 (41.2)	217 (34.1)	981 (44.3)	1123 (44.5)	1175 (35.8)	2486 (47.4)	5812 (40.7)
Black race, n (%)	1151 (18.2)	1271 (45.9)	68 (10.7)	1073 (48.5)	3 (0.1)	3287 (100)	1419 (27.0)	6019 (42.1)
BMI, kg/m^2								
Mean (SD)	28.7 (5.7)	29.3 (6.9)	26.9 (4.3)	29.6 (6.8)	27.6 (5.5)	31.8 (7.3)	28.2 (5.5)	29.0 (6.2)
BMI categories, n (%)								
<18.5	44 (0.7)	20 (0.7)	6 (0.9)	26 (1.2)	27 (1.1)	16 (0.5)	50 (1.0)	171 (1.2)
18.5 to <25	1665 (26.3)	776 (28)	221 (34.7)	572 (25.8)	872 (34.5)	470 (14.3)	1538 (29.3)	3823 (26.7)
25 to <30	2464 (38.9)	936 (33.8)	285 (44.7)	740 (33.4)	930 (36.8)	1064 (32.4)	2021 (38.5)	5161 (36.1)
30 to <35	1367 (21.6)	536 (19.3)	96 (15.1)	460 (20.8)	447 (17.7)	826 (25.1)	1066 (20.3)	2985 (20.9)
≥35	796 (12.6)	503 (18.2)	29 (4.6)	416 (18.8)	248 (9.8)	911 (27.7)	574 (10.9)	2153 (15.1)
Systolic BP, mean (SD), mmHg	127.2 (18.9)	115.9 (15)	131.1 (20)	123.6 (17.6)	115.6 (14.2)	126.5 (16.7)	126.4 (21.2)	126.3 (16.5)
Comorbidities								
Hypertension, n (%)	2565 (40.5)	369 (13.3)	318 (49.9)	299 (13.5)	284 (11.2)	1424 (43.3)	1688 (32.2)	6161 (43.1)
Diabetes, n (%)	687 (10.8)	152 (5.5)	61 (6.4)	181 (8.2)	52 (2.1)	472 (14.4)	543 (10.3)	1963 (13.7)
Smoking, n (%)	872 (13.8)	512 (18.5)	32 (5.0)	596 (26.9)	264 (10.5)	396 (12.0)	686 (13.1)	2057 (14.4)
Serum cholesterol								
Total, mean (SD), mg/dL	201.0 (30.2)	189.0 (28.9)	203.8 (35.4)	186.2 (32.9)	191.8 (28.6)	198.1 (31.8)	196.7 (29.9)	200.9 (31.5)
HDL, mean (SD), mg/dL	51.2 (16.6)	54.5 (15.8)	55.7 (14.5)	49.1 (13.8)	60.2 (17.3)	52 (14.4)	51.2 (14.8)	53.7 (16.4)
Waist circumference, mean (SD), cm	101.5 (14.8)	91.1 (14.9)	91.8 (12.6)	99 (16.3)	95.9 (14.6)	100.1 (16.4)	97.6 (14.4)	94.1 (15.2)
High sensitivity C-reactive protein, mean (SD) mg/L	2.5 (1.1, 5.5)	1.1 (0.5, 3.1)	1.6 (0.8, 2.8)	2.7 (1.2, 6.4)	1.0 (0.4, 2.6)	0.3 (0.1, 0.6)	1.9 (0.8, 4.3)	2.2 (1.0, 5.1)
Follow-up, median (IQR), years	15.8 (13.1-16.7)	9.0 (8.7-9.3)	7.7 (3.5-12.4)	11.4 (10.9-11.8)	10.6 (9.3-11.6)	11.8 (10.8-12.6)	13.0 (11.0-13.7)	9.0 (5.7-10.9)

© 2020 Khera R et al. *JAMA Network Open*.

Predicted 10-year ASCVD risk, mean (SD), %	11.9 (9.6)	2.5 (3.2)	16.7 (10.6)	4.2 (6.3)	2.1 (2.9)	9.1 (9.6)	12.9 (13.0)	14.3 (12.9)
Observed 10-year ASCVD event rates (%)	819 (12.9)	58 (2.1)	401 (62.9)	119 (5.4)	39 (1.6)	221 (6.7)	504 (9.6)	1548 (10.8)
CHD death, n (%)	153 (1.6)			429 (1.6)				
Non-fatal myocardial infarction, n (%)	706 (7.2)			821 (3.0)				
Fatal or non-fatal stroke, n (%)	421 (4.3)			1179 (4.3)				

Abbreviations: ASCVD – atherosclerotic cardiovascular disease, BP – blood pressure, CHD – coronary heart disease, cm – centimeters, HDL – high-density lipoprotein, hs – high-sensitivity, IQR – interquartile range, mg/dL – milligram per deciliter, mmHg – millimeters of mercury, SD – standard deviation

© 2020 Khera R et al. *JAMA Network Open*.

eTable 2. Number of Events and Individuals Across Obesity Classes by Body Mass Index for Groups Based on Their Estimated 10-Year Risk of Atherosclerotic Cardiovascular Disease from the Pooled Cohort Equations

**Predicted risk, %**	**Body Mass Index (kg/m^2)**									
	**<18.5**		**18.5 to <25**		**25 to <30**		**30 to <35**		**≥35**	
	Events	N	Events	N	Events	N	Events	N	Events	N
**<5**	4	320	188	9482	238	10136	148	5510	130	4586
**5-7.5**	14	82	166	1968	210	3174	128	1866	58	1314
**7.5-20**	30	186	724	5262	1238	8932	680	5360	460	3654
**≥20**	26	132	746	3162	1234	4960	672	2830	324	1706

© 2020 Khera R et al. *JAMA Network Open*.

eTable 3. Likelihood Ratio Statistic for Model Performance for the Pooled Cohort Equations, Overall and Among Strata Based on Obesity Class and Cohorts

Group	Likelihood ratio		
	**Overall**	**ARIC, CARDIA, CHS**	**DHS, MESA, FHS, JHS, REGARDS**
Overall	1448.8785	384.3265	1086.3575
BMI category (BMI range, kg/m^2)			
Underweight (<18.5)	22.6268	3.4569	19.5861
Normal (18.5 to <25)	389.4362	69.0026	324.4546
Overweight (25 to <30)	566.6078	169.6491	410.585
Mild Obesity (30 to <35)	278.0843	78.5087	200.4981
Moderate to Severe Obesity (≥35)	169.5841	52.5395	121.7949

© 2020 Khera R et al. *JAMA Network Open*.

eTable 4. Calibration of the Pooled Cohort Equations Stratified by Sex and Race

Group	E/O ratio			
	**Men**	**Women**	**Black**	**Non-Black**
Overall	1.279 (1.228, 1.332)	1.151 (1.108, 1.197)	1.273 (1.218, 1.328)	1.179 (1.14, 1.228)
BMI category (BMI range, kg/m^2)				
Underweight (<18.5)	1.832 (0.965, 3.426)	0.857 (0.625, 1.151)	1.841 (0.978, 3.339)	0.866 (0.637, 1.234)
Normal (18.5 to <25)	1.244 (1.148, 1.337)	1.174 (1.073, 1.274)	1.170 (1.054, 1.304)	1.234 (1.154, 1.312)
Overweight (25 to <30)	1.211 (1.146, 1.284)	1.112 (1.046, 1.191)	1.253 (1.163, 1.352)	1.130 (1.070, 1.202)
Mild Obesity (30 to <35)	1.391 (1.277, 1.522)	1.094 (1.006, 1.205)	1.309 (1.2, 1.428)	1.182 (1.087, 1.287)
Moderate to Severe Obesity (≥35)	1.521 (1.315, 1.766)	1.296 (1.174, 1.432)	1.375 (1.247, 1.518)	1.33 (1.17, 1.512)

Data represent mean expected to observed (E/O) risk ratio with corresponding 95% confidence intervals. A ratio of 1 indicates perfect calibration across the full spectrum of risk, and ratios above 1 and below 1 indicate average over- and under-estimation of risk, respectively.

© 2020 Khera R et al. *JAMA Network Open*.

eTable 5. Sensitivity Analyses After Stratifying by Median Age of the Participants at First Visit and Excluding Individuals With Subsequent Statin/lipid-lowering Therapy Use

**Age of participants at first visit**	**Above median**		**Below median**	
	**C-statistic**	**E/O ratio**	**C-statistic**	**E/O ratio**
Overall	0.666 (0.656, 0.677)	1.203 (1.167, 1.245)	0.766 (0.749, 0.784)	1.207 (1.139, 1.288)
BMI category (BMI range, kg/m^2)				
Underweight (<18.5)	0.813 (0.778, 0.848)	0.965 (0.740, 1.328)	0.905 (0.839, 0.972)	0.692 (0.415, 1.79)
Normal (18.5 to <25)	0.721 (0.680, 0.723)	1.193 (1.124, 1.274)	0.763 (0.733, 0.793)	1.206 (1.05, 1.425)
Overweight (25 to <30)	0.651 (0.549, 0.752)	1.158 (1.104, 1.213)	0.756 (0.716, 0.795)	1.166 (1.049, 1.284)
Mild Obesity (30 to <35)	0.673 (0.657, 0.690)	1.237 (1.145, 1.324)	0.674 (0.654, 0.694)	1.205 (1.068, 1.365)
Moderate to Severe Obesity (≥35)	0.653 (0.622, 0.685)	1.355 (1.231, 1.489)	0.651 (0.627, 0.675)	1.328 (1.176, 1.529)
**Excluding interim statin/lipid lowering therapy use**				
	**C-statistic**	**E/O ratio**		
Overall	0.762 (0.754, 0.770)	1.231 (1.202, 1.276)		
BMI category (BMI range, kg/m^2)				
Underweight (<18.5)	0.774 (0.709, 0.839)	1.039 (0.819, 1.428)		
Normal (18.5 to <25)	0.780 (0.766, 0.795)	1.226 (1.162, 1.315)		
Overweight (25 to <30)	0.769 (0.756, 0.781)	1.197 (1.143, 1.26)		
Mild Obesity (30 to <35)	0.739 (0.720, 0.759)	1.238 (1.16, 1.329)		
Moderate to Severe Obesity (≥35)	0.741 (0.716, 0.765)	1.355 (1.252, 1.504)		

Data represent C-statistic and mean expected to observed (E/O) risk ratio with corresponding 95% confidence intervals. A ratio of 1 indicates perfect calibration across the full spectrum of risk, and ratios above 1 and below 1 indicate average over- and under-estimation of risk, respectively.

© 2020 Khera R et al. *JAMA Network Open*.

eTable 6. Association of Obesity-Specific Measures With ASCVD Events in Race- and Sex-Specific Subgroups

Obesity-specific measure	Men	Women	Black	Non-Black
BMI	0.98 (0.93, 1.04)	0.99 (0.94, 1.06)	0.95 (0.89, 1.02)	1.02 (0.97, 1.07)
Waist circumference	1.03 (0.98, 1.08)	1.10 (1.04, 1.17)	1.04 (0.98, 1.10)	1.09 (1.03, 1.15)
hs-CRP	1.06 (1.04, 1.09)	1.07 (1.02, 1.11)	1.06 (1.02, 1.10)	1.09 (1.06, 1.13)
**All-together**				
BMI	0.86 (0.77, 0.95)	0.77 (0.69, 0.86)	0.79 (0.71, 0.89)	0.85 (0.77, 0.93)
waist circumference	1.16 (1.06, 1.28)	1.33 (1.21, 1.48)	1.23 (1.11, 1.36)	1.23 (1.12, 1.36)
hs-CRP	1.06 (1.04, 1.09)	1.06 (1.02, 1.11)	1.06 (1.02, 1.10)	1.09 (1.06, 1.13)
**Model discrimination (C-statistic and 95% CI)**				
Overall	0.740 (0.720, 0.760)	0.770 (0.752, 0.788)	0.748 (0.725, 0.771)	0.774 (0.758, 0.790)
Overall + BMI	0.741 (0.721, 0.760)	0.770 (0.752, 0.788)	0.748 (0.726, 0.771)	0.774 (0.758, 0.790)
Overall + waist circumference	0.739 (0.720, 0.759)	0.768 (0.750, 0.786)	0.747 (0.724, 0.770)	0.772 (0.756, 0.789)
Overall + hs-CRP	0.742 (0.722, 0.762)*	0.771 (0.753, 0.789)	0.749 (0.726, 0.771)	0.776 (0.760, 0.792)*
Overall + BMI + waist circumference + hs-CRP	0.742 (0.722, 0.762)*	0.768 (0.750, 0.786)	0.745 (0.727, 0.772)*	0.774 (0.757, 0.790)

Data represent the hazard ratio (95% CI) for each obesity-specific measure (per 1-standard deviation change in the exposure) in a model with the standard PCE as a predictor and incident ASCVD as an outcome, and the model discrimination (C-statistic) with adding each of these obesity specific measures to a standard PCE based risk-prediction model.*P<0.05 when c-statistic compared with c-statistic from standard PCE model

© 2020 Khera R et al. *JAMA Network Open*.

eTable 7. Association Between Obesity-specific Measures and Incidence of ASCVD in Models That Account for Competing Risk of Non-ASCVD Mortality*

**Obesity-specific measures**	**Hazard ratio (95% confidence interval), for one standard deviation change in measure**
Body mass index	1.00 (0.96, 1.04)
Waist circumference	1.07 (1.03, 1.12)
High-sensitivity C-reactive protein	1.04 (1.02, 1.06)
Combined model	
Body mass index	0.84 (0.78, 0.90)
Waist circumference	1.23 (1.15, 1.32)
High-sensitivity C-reactive protein	1.04 (1.02, 1.06)

*Models with variables included in the pooled cohort equations refit to our data set.

© 2020 Khera R et al. *JAMA Network Open*.

eTable 8. Association of Obesity-Specific Measures With ASCVD Events With Lipid Components Removed From the PCE

Obesity-specific measure	Overall	ARIC, CARDIA, CHS	DHS, FHS, JHS, MESA, REGARDS
**Hazard Ratio and 95% CI**			
BMI	1.006 (1.000, 1.012)	1.012 (1.001, 1.023)	1.003 (0.996, 1.011)
Waist circumference	1.007 (1.004, 1.009)	1.006 (1.002, 1.011)	1.007 (1.004, 1.010)
hsCRP	1.011 (1.008, 1.014)	1.020 (1.013, 1.027)	1.009 (1.005, 1.013)
**Model discrimination (C-statistic and 95% CI)**			
Overall (standard PCE components)	0.760 (0.753, 0.767)	0.758 (0.746, 0.770)	0.761 (0.752, 0.770)
Overall + BMI*	0.765 (0.757, 0.772)	0.760 (0.748, 0.772)	0.768 (0.758, 0.777)
Overall + waist circumference*	0.763 (0.755, 0.770)	0.756 (0.744, 0.768)	0.767 (0.757, 0.776)
Overall + hsCRP*	0.767 (0.759, 0.774)	0.760 (0.748, 0.772)	0.770 (0.760, 0.779)
Overall + BMI + waist circumference + hsCRP*	0.764 (0.757, 0.772)*	0.754 (0.742, 0.767)*	0.770 (0.761, 0.780)*

Data represent the hazard ratio (95% CI) for each obesity-specific measure (per 1-standard deviation change in the exposure) in a model with the non-lipid PCE covariates as predictors and incident ASCVD as an outcome, and the model discrimination (c-statistic) with adding each of these obesity specific measures to a PCE based risk-prediction model without lipid components. BMI = body mass index; hsCRP = high sensitivity C-reactive protein

*P>0.05 when c-statistic compared with c-statistic from standard PCE model

© 2020 Khera R et al. *JAMA Network Open*.

eTable 9. Net Reclassification Improvement With Addition of Obesity-Specific Makers to the PCE Recalibrated to the Data Threshold of risk set at 7.5%.

	Increased Risk	Decreased Risk	Net Correctly Reclassified	NRI	NRI p-value
**PCEs + Body mass index**					
Event	10	10	0		
No event	70	50	0.000	0.000	0.667
**PCEs + Waist circumference**					
Event	115	95	0.001		
No event	735	805	0.000	0.002	0.072
**PCEs + High-sensitivity C-Reactive Protein**					
Event	150	125	0.002		
No event	1025	1245	0.001	0.003	0.007
**PCEs + All three measures**					
Event	235	240	0.000		
No event	1495	2035	0.003	0.003	0.031

© 2020 Khera R et al. *JAMA Network Open*.
